# A Tryptophan-Deficient Diet Induces Gut Microbiota Dysbiosis and Increases Systemic Inflammation in Aged Mice

**DOI:** 10.3390/ijms22095005

**Published:** 2021-05-08

**Authors:** Ibrahim Yusufu, Kehong Ding, Kathryn Smith, Umesh D. Wankhade, Bikash Sahay, G. Taylor Patterson, Rafal Pacholczyk, Satish Adusumilli, Mark W. Hamrick, William D. Hill, Carlos M. Isales, Sadanand Fulzele

**Affiliations:** 1 Department of Medicine, Augusta University, Augusta, GA 30912, USA; IYUSUFU@augusta.edu (I.Y.); KDING@augusta.edu (K.D.); GPATTERSON1@augusta.edu (G.T.P.); 2 Department of Cell Biology and Anatomy, Augusta University, Augusta, GA 30912, USA; KATSMITH4@augusta.edu (K.S.); mhamrick@augusta.edu (M.W.H.); 3 Department of Pediatrics, College of Medicine, University of Arkansas for Medical Sciences (UAMS), Little Rock, AR 72202, USA; UWankhade@uams.edu; 4 Arkansas Children Nutrition Center, Arkansas Children’s Research Institute, Little Rock, AR 72202, USA; 5 Department of Infectious Diseases and Immunology, University of Florida, Gainesville, FL 32608, USA; sahayb@ufl.edu; 6 Georgia Cancer Center, Augusta University, Augusta, GA 30902, USA; RPACHOLCZYK@augusta.edu; 7 Department of Pathology, University of Notre Dame, Notre Dame, IN 46556, USA; sadusumi@nd.edu; 8 Institute of Healthy Aging, Augusta University, Augusta, GA 30912, USA; 9 Department of Pathology, Medical University of South Carolina, Charleston, SC 29403, USA; hillwi@musc.edu; 10 Ralph H Johnson Veterans Affairs Medical Center, Charleston, SC 29403, USA

**Keywords:** tryptophan, systemic inflammation, dysbiosis, gut, microbiota

## Abstract

The gut microflora is a vital component of the gastrointestinal (GI) system that regulates local and systemic immunity, inflammatory response, the digestive system, and overall health. Older people commonly suffer from inadequate nutrition or poor diets, which could potentially alter the gut microbiota. The essential amino acid (AA) tryptophan (TRP) is a vital diet component that plays a critical role in physiological stress responses, neuropsychiatric health, oxidative systems, inflammatory responses, and GI health. The present study investigates the relationship between varied TRP diets, the gut microbiome, and inflammatory responses in an aged mouse model. We fed aged mice either a TRP-deficient (0.1%), TRP-recommended (0.2%), or high-TRP (1.25%) diet for eight weeks and observed changes in the gut bacterial environment and the inflammatory responses via cytokine analysis (IL-1a, IL-6, IL-17A, and IL-27). The mice on the TRP-deficient diets showed changes in their bacterial abundance of Coriobacteriia class, *Acetatifactor* genus, Lachnospiraceae family, *Enterococcus faecalis* species, *Clostridium* sp genus, and *Oscillibacter* genus. Further, these mice showed significant increases in IL-6, IL-17A, and IL-1a and decreased IL-27 levels. These data suggest a direct association between dietary TRP content, the gut microbiota microenvironment, and inflammatory responses in aged mice models.

## 1. Introduction 

Balanced macronutrients (fats, carbohydrates, and proteins), micronutrients (minerals and trace elements), and vitamins in our diets are important for maintaining healthy physiological systems. However, poor nutrition is commonly observed in older populations and associated with chronic disease conditions, such as impaired digestive health, a decline in cognitive function, and a compromised immune system [1]. Interestingly, studies have shown that diet alone could potentially have therapeutic effects in patients suffering from chronic inflammatory diseases (such as inflammatory bowel disease) [2]. Thus, a better understanding of the relationship between the diet and specific disease processes could have a significant impact on healthy lifespan in the aging population. 

Amino acids (AA), the building blocks for protein synthesis, and their many metabolites are particularly crucial for growth, reproduction, immunity, and whole-body homeostasis [3]. Tryptophan (TRP) is one of nine essential amino acids and is responsible for modulating physiological stress responses, neuropsychiatric health, oxidative systems, and the immune system [4]. TRP deficiency is associated with major depressive illness in both males and females, and, in select cases, these depressive symptoms can be reversed by TRP-rich diets [5,6]. Several studies have found that low-TRP diets can lead to growth retardation, impaired reticulocyte function, and fatigue resistance, emphasizing the importance of this amino acid in the human diet [7,8,9].

Studies have particularly highlighted the importance of TRP and its many metabolites in systemic and local intestinal inflammatory mechanisms. TRP deficiency has been shown to compromise immune response and impair disease resistance in teleost fish [4]. Furthermore, gut health and intestinal immunity require sufficient dietary TRP for efficient immunological response and intestinal homeostasis [10]. TRP metabolites (kynurenines, serotonin, and melatonin) and bacterial TRP metabolites (indole, indolic acid, skatole, and tryptamine) are known to support gut microbiota, microbial metabolism, the host’s immune system, and host–microbiota synergy [10]. In fact, disruptions in the gut lactobacillus strains responsible for TRP metabolism can lead to intestinal inflammation and colitis [11].

In this study, we aimed to better define tryptophan’s role in the immune response and its effect on the gut microbiome, specifically in relation to age. We fed aged mice TRP-deficient (0.1%), TRP-recommended (0.2%), and high-TRP (1.25%) diets for eight weeks and observed the responses to the gut bacterial environment and the inflammatory responses. We hypothesized that a TRP-deficient diet would exert a systemic pro-inflammatory response and alter gut bacteria homeostasis. 

## 2. Results

### 2.1. Gut Microbial Taxonomic Analysis

**Figure 1:**
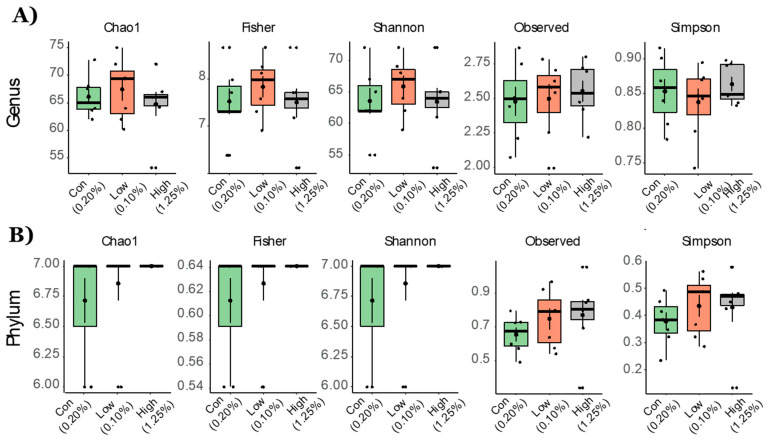
Alpha diversity measurements for aged mice fed with TRP-deficient and TRP-rich diets. α-diversity indices at the (**A**) genus and (**B**) phylum levels. Statistical differences between the group’s control diet (*n* = 7), TRP-deficient diet (*n* = 7), and TRP-rich diet (*n* = 7) were determined by Kruskal Wallis one-way ANOVA for doses of TRP. Data are expressed as mean ± SE and all comparisons at all indices were non-significant.

**Figure 2:**
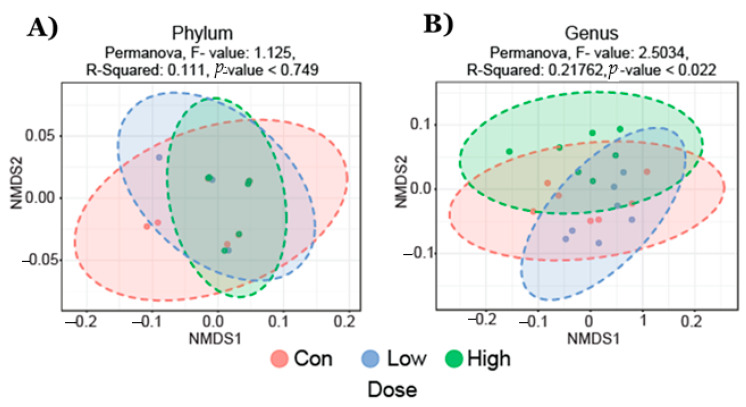
Non-metric multidimensional scaling (NMDS) plot of fecal bacterial community structures in animals fed with control diet (*n* = 7), TRP-deficient diet (*n* = 7), and TRP-rich diet (*n* = 7). Data showed no significant differences at (**A**) the phylum levels but formed distinct clusters at (**B**) the genus level specific to a TRP-deficient diet; (Permanova, F-value; 2.5034, R-squared; 0.21762, *p*-value < 0.022).

Fecal microbiota analysis was performed on three groups (21 total samples): control diet (*n* = 7), TRP-deficient diet (*n* = 7), and TRP-rich diet (*n* = 7). We analyzed the gut microbiota composition using the 16S rRNA amplicon sequencing of fecal contents and observed distinct differences in the microbial taxa associated with TRP dose variation. Microbiota diversity is typically described in terms of within (i.e., α) and between sample (i.e., β) diversities. α-diversity indices both at the phylum and genus level were not different between the groups of mice that were fed different doses of TRP (Figure 1). Non-metric multidimensional scaling (NMDS) ordination plots using a Bray–Curtis distancing matrix of β-diversity revealed significant differences at the genus levels (Dose, *p* < 0.05); however, at the phylum level there were no differences due to the TRP content in the diet (Figure 2).

### 2.2. Effect of TRP Supplementation on Taxonomical Differences

**Figure 3:**
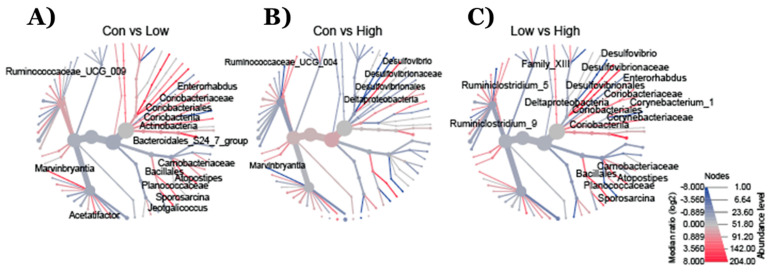
(**A**) Phylogenetic heat tree illustrates the differences in relative bacterial abundance between groups. The data show the changes in the bacterial families in mice fed (**A**) TRP-deficient/low compared to TRP-normal, (**B**) TRP-rich/high compared to TRP-normal, and (**C**) TRP-rich/high compared to TRP-deficient diets. Control diet (*n* = 7), TRP-deficient diet (*n* = 7), and TRP-rich diet (*n* = 7). Red nodes represent more abundant bacterial families, whereas the blue nodes represent less abundant bacterial families.

**Figure 4:**
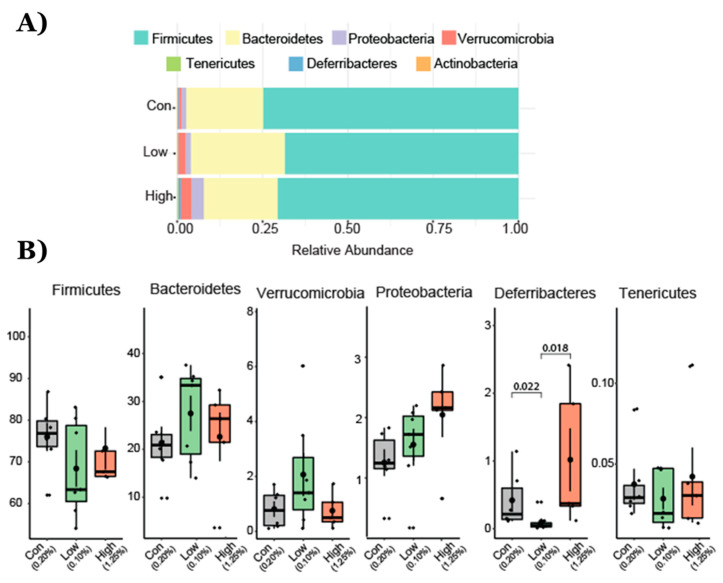
Operational taxonomical unit (OTU) abundance at the phylum level in animals fed with TRP-normal, TRP-deficient, and TRP-rich diets. (**A**) Relative increase in Verrucomicrobia and Bacteroideate and decrease in Firmicutes and Deferribacteres. (**B**) Box and whisker plots depict the operational taxonomical units (OTUs) from different bacterial phyla presented among three groups of mice. p values are shown where the differences were found to be significantly different from each other. Cont (control 0.2% TRP, *n* = 7), def/low (0.1% TRP, *n* = 7), and high/rich (control 1.25% TRP, *n* = 7).

**Figure 5:**
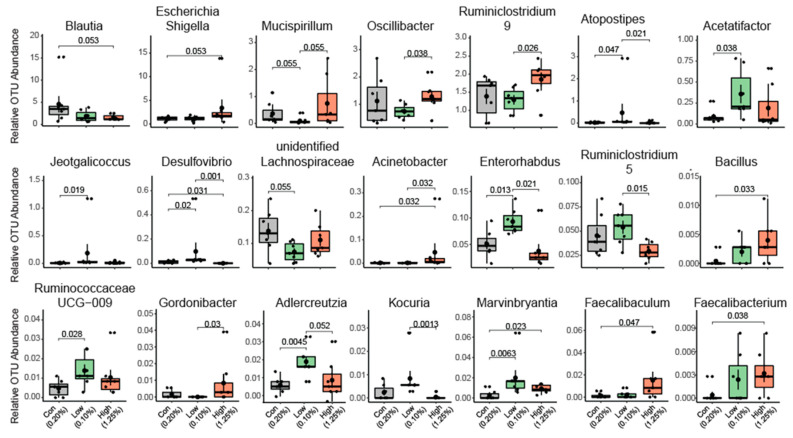
Change in composition of the gut microbiota at the genus level in animals fed with control diet (*n* = 7), TRP-deficient diet (*n* = 7), and TRP-rich diet (*n* = 7). Total of 21 genera were found to be different between at least one comparison (con vs. TRP-def/low, con vs. TRP-high, TRP-def/low vs. TRP high) The data represent OTUs of certain genera found to be different among the groups (data are expressed and mean +/− SE and l *p*-values were calculated using the Kruskal Wallis test).

The heat tree analysis leverages the hierarchical structure of taxonomic classifications to quantitatively (using the median abundance) and statistically (using the non-parametric Wilcoxon Rank Sum test) depict taxonomic differences between microbial communities or abundance profiles for a group. The Phylogenetic Heat Tree illustrates the differences in relative bacterial abundance between gut microbiota compositions in mice who were fed a diet with different TRP doses (Figure 3). Red colored nodes denote the enriched bacteria the comparison listed (Figure 3). Mainly Firmicutes and Bacteroidetes dominated gut microbiota composition at the phylum level. Other phyla such as Proteobacteria, Verrucomicrobia, Tenericutes, Deferribacteres, and Actinobacteria comprised the rest (Figure 4). Lower TRP supplementation reduced the Deferribacteres abundance, whereas higher supplementation not only restored but also increased the abundance (Figure 4). A trend for an increase in Proteobacteria abundance was seen in the mice who were fed the low- and high-dose TRP diets. At the genus level, out of 170 genera the Dunn test revealed significant groupwise differences in 21 genera (*p* < 0.05). Notably, Mucispirillum and lachnospiraceae bacteria went down with a low-TRP diet and were restored or increased in abundance on the higher TRP diet (Figure 5). The bacteria Acetatifactor, Enterorhabdus, and Adlercreutzia went up with a low-TRP diet and restored or decreased with a high-TRP diet (Figure 5).

### 2.3. Serum Cytokine Analysis

**Figure 6:**
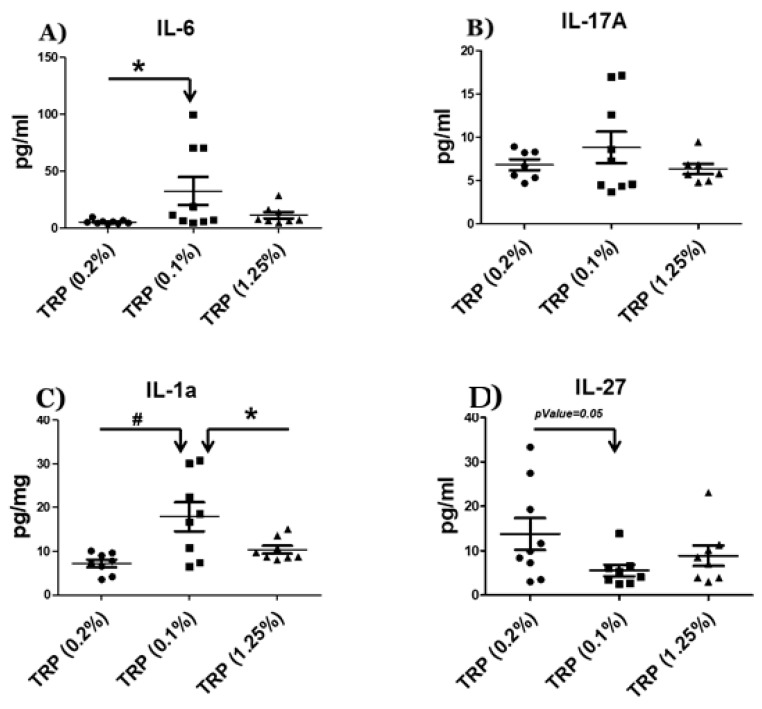
Serum cytokine levels in animals fed with a control diet, a TRP-deficient/low diet, and a TRP-rich diet. Serum was collected at the end of the experiment (week 8), followed by ELISA for immunoreactive (**A**) IL-6, (**B**) IL-17a (**C**) IL-1a, and (**D**) IL-27. Results are means ± SD (*n* = 7–9/per group). Data were analyzed by ANOVA followed by Bonferroni post hoc test or t-test (* *p* < 0.05, # *p* < 0.01).

It has been previously reported that an amino acid deficiency can induce systemic inflammation [12]. To assess the effects of the TRP-deficient vs. a TRP-rich diet on the systemic immune profile, we measured the pro-inflammatory cytokines IL-17A and IL-1a and the dual functioning pro- and anti-inflammatory cytokines IL-6 and IL-27 in the serum. We found that IL-6 was significantly (*p*-value = 0.04) elevated in the TRP-deficient diet, while a trend toward low IL-6 levels was found in the TRP-rich diet (compared to TRP-deficient diet). The pro-inflammatory cytokine IL-1a was significantly up-regulated compared to both the control (*p*-value = 0.008) and TRP-rich diets (*p*-value = 0.04). The anti-inflammatory cytokine IL-27 showed a significant (*p*-value = 0.05) decrease in the TRP-deficient diet compared to the control diet. The pro-inflammatory cytokine IL-17a showed a trend for up-regulation with the TRP-deficient diet compared to both the control and TRP-rich diets (Figure 6). Overall, the results revealed that the animals fed with the TRP-deficient diet had an elevated pro-inflammatory cytokine level compared to those fed a normal and a TRP-rich diet (Figure 6).

## 3. Discussion

The essential amino acid (AA) Tryptophan (TRP) plays a crucial role in regulating systemic immune responses and mental and gut health. However, an age-dependent relationship between TRP deficiency and gut microbiota health has not yet been reported. The present study explored this potential relationship between the gut microbiota and changes in the inflammatory milieu in aged mice after exposure to either a TRP-deficient (0.1%) or a TRP-rich (1.25%) diet for eight weeks. We found that the low-TRP diet animals exhibited altered bacterial composition in their gut microbiota compared animals fed with control and TRP-rich diets. Moreover, we also found that the low TRP diet-fed animals had an elevated level of systemic inflammation. 

Changes in the gut microbiota (dysbiosis) or a reduction in bacterial diversity can result in many intestinal diseases [13,14]. Our study found differences in the bacterial abundance of Coriobacteriia class, *Acetatifactor* genus, Lachnospiraceae family, *Enterococcus faecalis* species, *Clostridium* sp. genus, and oscillibacter genus in animals fed with TRP-deficient diets. Previously, this bacterial abundance/dysbiosis has been reported in a number of pathophysiological conditions [15,16]. *Enterorhabdus* genus is a member of the Actinobacteria phylum and Coriobacteriia class that has been associated with ileocecal mucosal inflammation in mice [1,2]. *Enterorhabdus mucosicola* and *Enterorhabdus caecimuris* species were both isolated from the ileocecal regions of mice suffering from colitis and intestinal inflammation [1,2]. An increased presence of *Enterorhabdus* genus is suggestive of promoting or is associated with mucosal inflammation in the GI tracts of mice. Our study found significant increases in *Enterorhabdus* genus abundance in TRP-deficient mice, suggesting that these animals might be under chronic inflammatory stress. 

Our study also found a significantly increased abundance of *Adlercreutzia genus* (Actinobacteria phylum) in the TRP-deficient mouse gut microbiota. Moon et al. (2018) found relatively low abundances of *Adlercreutzia* in diabetic women and increased plasma levels of TRP metabolites (e.g., kynurenine) [17]. Their findings suggest that gut-derived *Adlercreutzia* genus abundances are indirectly associated with increased TRP metabolism. These results are further supported by our finding that *Adlercreutzia* genus is correlated with TRP metabolites. We also noted more than a three-fold increase in *Acetatifactor* bacterium in TRP-deficient mice. Interestingly, *Acetatifactor muris* is associated with intestinal inflammation. Transplanting fecal microbiota rich in *Acetatifactor muris* into healthy wild-type mice induced colonic inflammation [15]. This bacterium’s significant increase in the gut microbiota could potentially promote intestinal inflammation and related adverse effects. Although our study found the *Acetatifactor* genus to increase, we found that the Lachnospiraceae family generally decreased significantly. Lachnospiraceae is a family of anaerobic, spore-forming bacteria that produce butyric acid, which is protective against colon cancer development in humans [16,18]. The general decrease in bacteria in the Lachnospiraceae family and increases in the *Acetatifactor* genus strongly indicated a systemic elevation of inflammation in the TRP-deficient group. 

It has been previously reported that *Clostridium* sp. metabolizes TRP and generates indoxyl sulfate (IS) and indole-3-propionic acid (IPA) [19]. Indoxyl sulfate is a toxic metabolite that enhances oxidative damage in intestinal epithelial cells and compromises the epithelial layer lining the intestines [20]. Conversely, IPA is a TRP metabolite that protects mice from dextran sodium sulfate (DSS)-induced colitis, suggesting that it has some anti-inflammatory role [21]. Previously, Konopelski et al. reported that rats on a TRP-free diet had a lower concentration of IPA in stool and blood [9]. In our study, we found *Clostridium* sp. to be significantly decreased in TRP-deficient mice. Our data indicated that TRP deficiency might have some protective as well as harmful pro-inflammatory effects via decreased *Clostridium* sp. abundance. *Clostridium* species are also known to regulate the critical neurotransmitter serotonin (5-HT) in the gut [22]. The human colon promotes 5-HT biosynthesis, which regulates many physiological processes, including neurotransmission, mood, sleep, memory, intestinal motility, and digestion [22]. Decreased *Clostridium* sp. may lead to lower levels of 5-HT, leading to depression, impaired digestions, and neuropsychiatric conditions. Similarly, the number of other gut bacterium significantly decreases in TRP-deficient groups such as *Oscillibacter valericigenes*, *Mucispirillum*, and *Blautia* genus. These gut bacteria play a significant role in maintaining human and animal gut microflora health [5,6,9,23]. Studies have found O. valericigenes to be significantly decreased in Crohn’s disease patients’ microflora, with elevated inflammation [23]. *Mucispirillum* genus bacterium encodes for proteins that resist the oxidative bursts associated with inflammatory states [5]. These bacterial species express superoxide reductase, catalase, cytochrome *c* oxidase, and rubrerythrin that utilize diverse reactions to neutralize reactive oxygen species [5,6]. The *Blautia* genus is known for its anti-inflammatory properties. For example, Jenq et al. demonstrated increased abundances of the *Blautia* genus with decreased graft-versus-host-disease mortality and improved survival after allogeneic bone marrow transplantation [9]. Their results suggest some level of anti-inflammatory properties mediated by the *Blautia* genus.

Based on dysbiosis in the gut microbiota of the TRP-deficient diet, we hypothesized that gut microbiota changes might induce the systemic release of cytokines. To investigate this, we measured the predominantly pro-inflammatory cytokines IL-17A and IL-1a and the dual functioning pro- and anti-inflammatory cytokines IL-6 and IL-27 in the serum of animals placed on the TRP-deficient diet. Our data showed significant increases in IL-6, IL-17A, and IL-1a, but a significant decrease in IL-27. It is interesting that the mice fed with a TRP rich diet presented with higher IL-27, a cytokine known to prevent IL-17 transcription. The production of IL-27 along with Aryl hydrocarbon receptor (AhR) activation helps in the generation of regulatory T cells in the gut [24]. IL-27 prevents excessive inflammation by controlling IL-17 responses by the generation of regulatory T cells. It also maintains the gut epithelial barrier, including the enhancement of indoleamine 2,3-dioxygenase (IDO1) expression [25]. The overall effect of TRP deficiency is geared toward a systemic pro-inflammatory state. The elevated level of systemic pro-inflammatory status in a TRP-deficient diet might be due to a combination of different reasons, such as (1) decreased levels of cellular TRP metabolites (Kynurenine pathway metabolites) and bacterial-derived TRP metabolites (indole, indolic acid, and tryptamine), (2) the dysregulation of AhR transcription factor, (3) increased oxidative stress due to the TRP deficiency diet and its metabolites (e.g., IPA), and/or (4) direct changes in the composition of the gut microbiota. The cellular and gut microbiota-derived TRP metabolites are endogenous ligands of AhR [26,27,28]. A low level of TRP in the diet may decrease endogenous ligands of AhR, leading to the dysregulation of transcription factor (AhR). It has been previously reported that the AhR plays a significant role in maintaining gut and systemic inflammation in humans and rodents [28,29]. Furthermore, a decrease in microbiota-derived TRP metabolites such as Indole-3-propionic acid (IPA) leads to the accumulation of reactive oxygen species (increased oxidative stress). IPA is a potent antioxidant known to play an important role in neuroprotection [30,31], anti-non-alcoholic steatohepatitis [32], and protection against radiation toxicity [33], and reduced the bacterial load in a mouse model of acute *M. tuberculosis* infection [34]. TRP metabolism signaling is complex; the endogenous metabolites of TRP (kynurenine pathway) are known to elevate the inflammatory process [35], whereas gut-derived indoles derivatives reveal an anti-inflammatory effect [21]. 

A balanced diet is required for minimizing age-associated diseases. The gastrointestinal (GI) system is a critical organ that is commonly compromised in the elderly population. Studies have postulated that gut microbiota’s dynamic changes in the aging population could modulate changes in immunity and cognitive functioning, thus contributing to certain diseases [36]. The bacterial gut composition in human and animal models has been shown to play a critical role in regulating intestinal conditions such as colitis and allergic diarrhea via regulatory T-cell (T-reg) modulation [10,11,37]. Most importantly, diet has been directly linked to microbiota composition in humans and rodents [10,11,13,14,36,37,38,39,40,41]. A further detailed investigation is needed to elucidate the relationships between the TRP metabolites, AhR signaling, and systemic inflammation in age-related pathophysiological conditions. 

## 4. Materials and Methods

### 4.1. Animal Study

All protocols were conducted by following the guidelines established by the Augusta University Institutional Animal Care and Use Committee (AU-IACUC, Protocol number: 2009-0065). Twenty-month-old male C57BL/6 mice were obtained from the aged rodent colony at the National Institute on Aging. The animals were fed either standard TRP (0.2%), low-TRP (0.1%) or high-TRP (1.25%) diets for eight weeks. TRP concentration was selected based on previously published data [42]. Diets were prepared by Envigo-Teklad (Madison, WI, USA) in consultation with their nutritionist and were isocaloric purified diets that contained all essential amino acids. Fecal samples were collected at the end of 8 weeks, and animals were euthanized using CO2 overdose followed by thoracotomy according to an AU IACUC-approved protocol.

### 4.2. Microbial Community Profiling Using 16S rRNA Amplicon Sequencing

The MO BIO PowerSoil DNA Isolation kit (Qiagen, Germantown, MD, USA) was used to isolate genomic DNA samples from the fecal samples of mice. Fecal contents were carefully added to 96-well plates with beads and recommended buffers. The plates were sealed, added to the MO BIO shaker, and shaken horizontally at 20 rpm for 20 min. The isolated genomic DNA was quantified with nanodrop and stored at −20 °C. The DNA samples were shipped to Novogene Corporation, Inc. (Durham, NC, USA), for 16S V3-V4 region amplicon sequencing.

### 4.3. Bioinformatics Analysis

BIOM files were analyzed using MicrobiomeAnalyst—a web-based tool for the statistical and visual analysis of microbiota data. Reads were initially denoized using filters with a minimum number of five reads in a minimum of one sample required to retain an OTU. Total read counts for the run were 754,355, with 35,921 per sample. Sample reads were normalized and rarefied to the minimum library, then data scaling (total sum scaling) was performed before the final analysis. Minimum read filtering was applied in MicrobiomeAnalyst for alpha and beta diversity calculations but was increased to a minimum of 10% prevalence with a count of 4 for differential abundance analysis. Low variance filter was set at 5% for the inter quartile range. Alpha diversity profiling and significance testing were conducted using one-way ANOVA at the genus and phylum taxonomic level. For beta diversity, permutational MANOVA (PERMANOVA) was used to compute groupwise differences. Differential abundance was calculated univariately. All *p*-values were calculated using the Kruskal Wallis test and the Dunn test unless stated otherwise. Microbiota figures (Figure 1, Figure 2, Figure 3 and Figure 4) were prepared using open licensed software R. 

### 4.4. Serum Cytokine Analysis

At the end of study, blood was drawn from animals by cardiac puncture. Levels of selected cytokines were measured in the serum by LEGENDplex Cytokines Detection (BioLegend, San Diego, CA, USA), as described by the manufacturer. The simultaneous quantification of cytokines in mouse sera was performed using the LEGENDplex mouse Inflammation Panel with V-bottom Plate (BioLegend Cat# 740446) according to the manufacturer’s instructions. In brief, samples were thawed completely, mixed, and centrifuged to remove particulates prior to use. To achieve measurement accuracy, samples were diluted 2-fold with assay buffer, and standards were mixed with Matrix C (BioLegend) to account for additional components in the serum samples. Standards and samples were plated with capture beads for IL-1α, IL-6, IL-17A, and IL-27 and incubated for 2 h at room temperature on a plate shaker (800 rpm). After washing the plate with wash buffer, detection antibodies were added to each well. The plate was incubated on a shaker for 1 h at room temperature. Finally, without washing, SA-PE was added and incubated for 30 min. Samples were acquired on the CytoFLEX flow cytometer (Beckman Coulter Life Sciences, Indianapolis, IN, USA). Standard curves and protein concentration were calculated using the R package DrLumi [43] installed on R 3.5.2 (https://www.r-project.org/, accessed on 7 May 2021). The limit of detection was calculated as an average of background samples plus 2.5 × SD. Assay and data calculations were performed using the Immune Monitoring Shared Resource (Augusta University).

### 4.5. Statistical Analysis

The results are shown as means ± standard deviations. GraphPad Prism 5 (La Jolla, CA, USA) was utilized to perform ANOVA with Bonferroni pair-wise comparison or unpaired *t*-tests as appropriate. A *p*-value of <0.05 was considered significant.

## Data Availability

The data that support the findings of this study are available from the corresponding author upon reasonable request.

## Abbreviations

GI	Gastrointestinal
AA	Amino acid
TRP	Tryptophan
IS	Indoxyl sulfate
IPA	IPA; Indole-3-propionic acid
IDO1	IDO1; Indoleamine 2,3-dioxygenase
AhR	AhR; Aryl hydrocarbon receptor
